# The implementation of Health in All Policies initiatives: a systems framework for government action

**DOI:** 10.1186/s12961-018-0295-z

**Published:** 2018-03-15

**Authors:** Ketan Shankardass, Carles Muntaner, Lauri Kokkinen, Faraz Vahid Shahidi, Alix Freiler, Goldameir Oneka, Ahmed M. Bayoumi, Patricia O’Campo

**Affiliations:** 1 Centre for Urban Health Solutions, Li Ka Shing Knowledge Institute, 209 Victoria Street, Toronto, ON M5B 1T8 Canada; 2 Dalla Lana School of Public Health, University of Toronto, 155 College Street, Toronto, ON M5T 3M7 Canada; 3 Department of Health Sciences, Wilfrid Laurier University, 75 University Ave W, Waterloo, ON N2L 3C5 Canada; 4 Bloomberg School of Nursing, University of Toronto, 155 College Street, Toronto, ON M5T 3M7 Canada; 5 Finnish Institute of Occupational Health, PL 40, 00251 Helsinki, Finland; 6 Department of Medicine and Institute of Health Policy, Management and Evaluation, University of Toronto, 155 College Street, Toronto, ON M5T 3M7 Canada

**Keywords:** System framework, Health equity, Health in all Policies, Intersectoral action

## Abstract

**Background:**

There has been a renewed interest in broadening the research agenda in health promotion to include action on the structural determinants of health, including a focus on the implementation of Health in All Policies (HiAP). Governments that use HiAP face the challenge of instituting governance structures and processes to facilitate policy coordination in an evidence-informed manner. Due to the complexity of government institutions and the policy process, systems theory has been proposed as a tool for evaluating the implementation of HiAP.

**Methods:**

Our multiple case study research programme (HiAP Analysis using Realist Methods On International Case Studies – HARMONICS) has relied on systems theory and realist methods to make sense of how and why the practices of policy-makers (including politicians and civil servants) from specific institutional environments (policy sectors) has either facilitated or hindered the implementation of HiAP. Herein, we present a systems framework for the implementation of HiAP based on our experience and empirical findings in studying this process.

**Results:**

We describe a system of 14 components within three subsystems of government. Subsystems include the executive (heads of state and their appointed political elites), intersectoral (the milieu of policy-makers and experts working with governance structures related to HiAP) and intrasectoral (policy-makers within policy sectors). Here, HiAP implementation is a process involving interactions between subsystems and their components that leads to the emergence of implementation outcomes, as well as effects on the system components themselves. We also describe the influence of extra-governmental systems, including (but not limited to) the academic sector, third sector, private sector and intergovernmental sector. Finally, we present a case study that applies this framework to understand the implementation of HiAP – the Health 2015 Strategy – in Finland, from 2001 onward.

**Conclusions:**

This framework is useful for helping to explain how, why and under what circumstances HiAP has been successfully and unsuccessfully implemented in a sustainable manner. It serves as a tool for researchers to study this process, and for policy-makers and other public health actors to manage this process.

## Background

By highlighting the importance of the structural determinants of health as root causes of health inequity, the 2008 final report by the Commission on the Social Determinants of Health encouraged broadening of the health promotion research agenda to include a focus on government policies and processes, alongside culture and societal values of populations [1].

Governments that apply the idea of Health in all Policies (HiAP) to strengthen health equity strive for durable and systematic approaches to pursuing intersectoral action that leads to healthy and equitable public policies. Thus, the implementation of HiAP typically involves instituting what Fafard refers to as “*integrated governance*” ([2], p. 2), in which governance structures and processes are used to facilitate policy coordination in an evidence-informed manner, sometimes guided by explicit long-term strategies and goals [3]. In this formulation, HiAP is not a specific approach (as presented more recently by WHO [4]), but rather a general concept that accommodates diverse initiatives, where “*HiAP initiatives may include multiple programmes or projects that are fostered across multiple sectors and multiple levels of government either directly or indirectly related to the original policy commitment*” ([5], p. 465). Thus, assessing the implementation of HiAP includes a focus on how sectors and approaches are coordinated and integrated over time.

As more governments have adopted HiAP approaches globally, there have been calls for greater monitoring to better understand how integrated governance can be used to meet health and equity objectives [4, 6–8]. The need for such attention stems from multiple challenges. First, the process of implementing HiAP can be challenging since policy-makers are not always experienced in working intersectorally. For example, long-standing government traditions of policy development and implementation within distinct organisational and professional silos can be hard to transform [9]. Second, there is often a lack of evidence about how to best facilitate successful HiAP implementation; most evidence is descriptive rather than analytic [10]. Third, since HiAP is a concept rather than a model, every HiAP initiative is uniquely designed and governed, and so it is challenging to understand how to translate studies of one case to others.

Bhatta notes that because “*it is much easier to set goals than to put them into action, implementation considerations tend to be less accounted for than the other processes in policy development*” ([11], p. 456). In the case of implementing HiAP, guidance has generally focused on technical challenges of policy implementation [12–15], whereas there are also political challenges to HiAP implementation given the focus on health equity improvement [16]. More fundamentally, there has been little application of theory to help test hypotheses about why certain implementation practices work in some settings, and what other contextual and collective factors influence implementation outcomes. Moreover, the complexity of understanding policy processes makes the generation of hypotheses difficult [17].

Systems theory has been proposed as a tool for evaluating health promotion activities, including those undertaken by governments [18, 19]. The use of systems theory implies that there is no simple approach to implementing HiAP; rather, implementation outcomes are mostly unpredictable given the complex inter-relationships between system components (e.g. sectors with competing priorities, political turnover and changing policy agendas). Yet, systems theory can offer a framework for understanding implementation phenomena, including where implementation is based on short-sighted thinking or where it goes wrong [19]. This approach has been used more extensively in understanding the management of healthcare organisations [20], and has even been associated with increased effectiveness of interventions in these settings [21]. Although there are some exceptions (e.g. [22]), systems theory has not been adequately integrated in the discourse about how to implement HiAP.

From the outset, our multiple case study research programme (HiAP Analysis using Realist Methods On International Case Studies – HARMONICS) has relied on systems theory to make sense of how and why the practices of policy-makers (including politicians and civil servants) from specific institutional environments (policy sectors) has either facilitated or hindered the implementation of HiAP [5]. To help other researchers learn from case studies of HiAP implementation, this article begins by providing a description of systems theory followed by a description of a system of 14 components within three subsystems of government, which we argue mainly explain implementation outcomes alongside some important extra-governmental influences. We then use a case study to demonstrate how to apply the systems theory framework, which involves the implementation of the Health 2015 Strategy in Finland from 2001 onward, conceptualised herein as a form of HiAP [23].

Social systems (i.e. systems composed of individuals and the relations between them) are difficult to quantify, which can hinder monitoring and evaluation. The framework we propose, along with other empirical analyses from HARMONICS (e.g. [24]), offers both theory and an analytic approach based on this theory. This approach allows the generation of hypotheses about the implementation process and the testing of these hypotheses by evaluating how well the theory explains how, why and under what circumstances HiAP has been successfully implemented in a sustainable manner. Policy-makers are of particular interest as individuals who are engaged in and possibly managing aspects of the government system of HiAP implementation. For example, the implementation strategies of policy-makers may affect how resources are distributed to lead and support implementation activities. Policy-makers can use this framework to understand their own government systems, and to anticipate the potential challenges and impacts of various strategies for HiAP implementation.

## A Systems Theory primer

Systems Theory emerged in the Twentieth century as a set of theories that encompassed multiple fields, including philosophy as well as basic and applied science (e.g. computing science) [25, 26]. It can be labelled a meta-theory in that Systems Theory searches for commonalities across biological, physical and social systems. In brief, it posits that the world is made of wholes (systems) whose properties (including emergent outcomes) cannot be explained by a simple accounting of its components [27]. Components are organised within subsystems that are inherently interdependent [28], so changes to one subsystem will affect others, sometimes in unexpected ways [26]. Emergent properties of a system are those not possessed by any single component of the system, and emergence refers to the process by which the system acquires these properties [29]. Such ontology leads to an epistemology that rejects both reduction (the exclusive study of the components of a system) and holism (the neglect of the components of a system).

To use an example from outside the domain of HiAP implementation, we may want to understand social cohesion in a neighbourhood (social) system. Studying only the psychology of neighbourhood residents would not give insight into inter-personal processes, such as reciprocity norms or exchange networks, which constitute important generative mechanisms to explain social cohesion. Mingers [27] describes generative mechanisms as the processes that keep a system in motion, which occur in relation to specific contextual conditions. Herein, the study of system components alone (e.g. social characteristics, such as the criminality of neighbourhood residents) cannot explain emergent properties of this system, like social cohesion, because they do not uncover the relations among components (e.g. norms of reciprocity) and the role of context.

Emergent properties arise as a consequence of interactions between system components, including feedback that changes system components and other outcomes of relevance [30, 31]. Systems can therefore operate in non-linear ways with seemingly obvious solutions sometimes worsening a problem ([32], p. 19). Systems and subsystems have boundaries that are permeable, so that changes to systems in the external environment of these domains may influence how and why the system works [26, 33].

As described below, this systems framework focuses on how certain governance structures, processes and strategies can help to set the agenda and build capacity for HiAP implementation to facilitate sustainable initiatives. In doing so, this conceptual framework includes a series of subsystems, components and external influences that can inform hypotheses about how to explain and anticipate HiAP implementation outcomes in different settings.

## HiAP implementation within a government system: the HARMONICS approach

Since HiAP initiatives are typically mandated to implement a variety of health equity interventions across multiple policy sectors and multiple geographic levels of government over time (e.g. [34]), we view implementation as requiring an on-going adaptive process for governments in terms of their governance structures and strategies, similar to what Hummelbrunner calls an “*open change process*” ([35], p. 395). Thus, the systems framework described below is intended as a tool for researchers to study this process and for policy-makers and other public health actors to manage it.

This framework was developed by our team iteratively over the course of many discussions between 2015 and 2016, with a focus on articulating the most important and unique facets of government systems relevant to the implementation of HiAP. These discussions were informed by (1) several existing theories about policy implementation (e.g. [2, 3]), including our own initial frameworks about the implementation of HiAP [3, 5], and (2) a wide range of evidence on the topic of implementing intersectoral action for health equity [10], including our analysis of three case studies of HiAP implementation in Sweden, Quebec and South Australia [3, 24, 36]. Importantly, we also apply a realist scientific approach to our case studies, which is an ontology that has focused our work on understanding the oft-hidden generative mechanisms that explain implementation outcomes (described further below) [5].

**Fig. 1:**
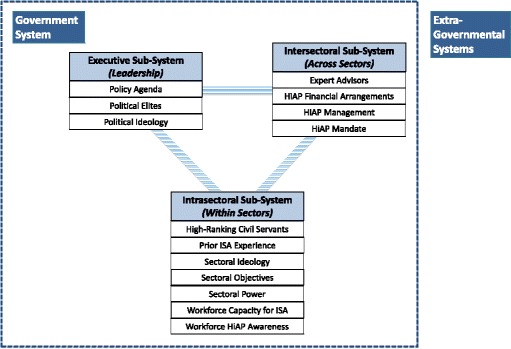
A systems framework depicting 14 components within three government sub-systems involved in HiAP implementation

The framework below presents HiAP implementation as a process involving interactions between system components and subsystems in our model that leads to the emergence of both favourable and unfavourable implementation outcomes, as well as effects on the system components themselves (i.e. feedback). Figure 1 and Table 1 outlines our systems framework for HiAP implementation in terms of a series of components pertaining to three subsystems, including the executive (heads of state and their appointed political elites), intersectoral (the milieu of policy-makers and experts working with governance structures related to HiAP) and intrasectoral (policy-makers within policy sectors).

**Table 1:** Definition of sub-systems and system components

Executive Subsystem: The processes of government responsible for the creation and implementation of legislative mandates related to the implementation of HiAP initiatives.	*Policy agenda*: The finite set of social and political issues upon which governments act on at a given point in time, which will be shaped by the party organisation(s) who control the government and influenced by extra-governmental factors, and which have implications for the priority of health equity initiatives like HiAP.
	*Political elites*: Actors who, by virtue of some form of formal authority within the government, exhibit a consistent and substantial level of control over the political process and its outcomes, including as they relate to the implementation of HiAP initiatives.
	*Political ideology*: The cluster of ideas, beliefs, values and attitudes that constitute the normative lens through which political elites and party leaders interpret and act upon social and political issues. For example, they have political interests in relation to their opinions about ‘what ought to be*’* when it comes to developing or modifying policies during the implementation of HiAP initiatives. Those opinions may, in turn, be shaped by their experiences and understanding of the real world, and by their more fundamental worldviews and ideological subscriptions, e.g. the role of state or the primacy of individual responsibility.
Intersectoral Subsystem: The processes of government that facilitate the horizontal and vertical coordination of the HiAP policy agenda across various sectors of the government and with extra-governmental partners.	*Expert advisors*: Expert individuals (often from outside of government) who are formally consulted in planning and executing the implementation of HiAP initiatives. Expert advisors are a type of policy elite, i.e. they have influence over the policy process.
	*HiAP financial arrangements*: Financial arrangements that dictate the magnitude, distribution and sources of funding available for the implementation of HiAP initiatives.
	*HiAP management*: The set of technical processes through which governments generate institutional capacity for implementation of HiAP initiatives.
	*HiAP mandate:* Official legislation and formal strategies containing specific instructions for the implementation of HiAP initiatives (e.g. policy goals, division of responsibility, allocation of resources, processes for monitoring and enforcement), which may change or grow in number over the period of implementation (see Freiler et al. 2013 for additional information).
Intrasectoral Subsystem: The processes of government that facilitate activities such as the pursuit of sectoral objectives, which may be affected by the implementation of HiAP initiatives.	*High-ranking civil servants:* Bureaucrats who may have authority over the policy process delegated to them by political elites. High-ranking civil servants are a type of policy elite, i.e. they have influence over the policy process, and may be particularly engaged in the technical aspects of implementing HiAP initiatives.
	*Prior experience with intersectoral action (ISA):* A history of working intersectorally on shared policy objectives, which may influence how the implementation of related initiatives, such as HiAP, occurs.
	*Sectoral ideology:* The cluster of ideas, beliefs, values and attitudes that constitute the normative lens through which policy-makers within a given sector interpret and act upon social and political issues such as health equity, and which may vary given sectoral objectives (e.g. healthcare, population health, economic growth, engineering), i.e. a worldview.
	*Sectoral objectives*: Goals and motivations of policy sectors, often delivered through a formal mandate from the executive, which may be affected by a government’s implementation of HiAP initiatives.
	*Sectoral power*: The formal authority allocated to policy sectors in government mandates, indicated in absolute (e.g. directives, budget size) and relative terms across sectors (e.g. relative budget size). Intersectoral coordination of policies in the process of implementing HiAP initiatives may be shaped by the power of the sectors involved.
	*Workforce capacity for ISA*: The extent of expertise among human resources with tools and processes and workforce size dedicated to implementing HiAP initiatives, enabling feasibility (see Freiler et al. 2013 for additional information).
	*Workforce HiAP awareness*: An understanding of the need and reasons for an intersectoral approach to address health equity, as part of the process of agenda setting and, ultimately, buy-in for the implementation of HiAP initiatives (see Freiler et al. 2013 for additional information).
Extra-governmental systems: Systems outside of government that can influence HiAP implementation, including as organisations and individuals become partnered to the implementation of HiAP; for example, by participating in planning or executing intersectoral action or in being the subject of some attendant regulatory action. There are also likely to be more indirect influences, such as policy entrepreneurs who advocate or lobby for influence over the implementation process, and cross-national policy and agenda-setting frameworks. Finally, at the global and local levels, there are research programmes and knowledge hubs producing information to support implementation.	

The implementation of HiAP often requires policy coordination across multiple government levels or systems (e.g. national, provincial/state, regional and local), as well as with other systems outside of government that affect health equity (that do not necessarily look like Fig. 1). Therefore, we also include a role for extra-governmental systems, which can influence the implementation of HiAP in many ways (described further below).

In HARMONICS, systems theory has been useful for testing hypotheses and explaining phenomena about two favourable outcomes of the implementation process, namely the acceptability (i.e. are sectors willing to collaborate on health and equity?) and feasibility (i.e. do sectors have the capacity to effectively collaborate on health and equity?) of HiAP implementation for partners in diverse policy sectors related to the ongoing process of implementation [37]. Acceptability and feasibility are favourable inasmuch as they contribute to more sustainable implementation of HiAP and they may also lead to stronger types of HiAP.

For example, acceptability and feasibility may facilitate a broader inclusion of policy sectors that participate in HiAP over time, reflecting both a larger number of sectors and greater diversity among those sectors, e.g. including social and economic-oriented sectors. There may also be stronger equity interventions (i.e. interventions that are more upstream), as reflected by the values and ideas implicit in the theory of change for resulting health equity interventions.

## Case study: The implementation of Health 2015 in Finland

Below, we demonstrate the value of using the systems framework of HiAP implementation (system components from Fig. 1 and Table 1 are *italicised*) to organise information about a particular policy issue and outcome in the implementation of Health 2015 in Finland. Following the case description, we offer some reflections on generative mechanisms and next steps for understanding the outcomes of the case.

Our case study draws evidence from a realist explanatory case study of this project [23], including the analysis of peer-reviewed and grey literature, government reports and key informant interviews with 10 policy-makers involved in the implementation of Health 2015. More details about our methodology are available in Shankardass et al. [5].

### Background of intersectoral action (ISA) for health equity in Finland

In the early 1970s, it was recognised by public officials that the health of Finland’s population was lagging behind economic progress [38]. As a result, the improvement of population health became a political priority of the Finnish government (*policy agenda*). Since that time, policy sectors have been growing their *(prior) experience with ISA*. In 1972, the government adopted a public health law that introduced a framework for intersectoral collaboration between the health sector and other government sectors, as well as non-governmental organisations and the private sector, to reduce cardiovascular disease in the population. In 1986, a national Health For All strategy was implemented to encourage broader efforts towards ISA for health. Following an evaluation conducted by WHO experts [39], a revised strategy was adopted in 1993 to accommodate a greater focus on health equity [40]. Overall, there was a long history of prior experience with ISA before Health 2015 was adopted, which would have created some institutional awareness about the problem of health equity and the need for ISA to address this problem.

At the turn of the millennium, as the term of the Health For All strategy was ending, Health 2015 was adopted as a long-term strategy for addressing health equity using ISA (*HiAP mandate*; see [41] for more details). As part of the implementation of the Health 2015, Finland’s Ministry of Social Affairs and Health developed guidelines to direct other sectors to consider using Health Impact Assessments to contribute to the evaluation of key policy decisions – a form of *HiAP management*. This was a process suggested by WHO and the EU (*environmental influences – supranational political organisations*). No laws were introduced alongside these guidelines to make participating in Health 2015 mandatory, as were passed in the case of Quebec’s revised Public Health Bill [41], another example of a HiAP initiative.

Our case study focuses on a single policy issue that arose during the implementation of Health 2015 concerning the government’s decision-making about whether to reform national alcohol policy (*policy agenda*) following the introduction of Estonia to the EU in 2004 (*external influences – supranational political organisation*), in the context of a Health Impact Assessment that was conducted on this issue.

Since the 1970s, the cost of alcohol in Finland had been regulated by a high tax rate that discouraged the consumption of alcohol on the one hand and provided a convenient source of tax revenue on the other [42]. From the point of view of maintaining high taxes, the Finnish government considered this arrangement to be viable as long as Sweden and Denmark acted as buffers between Finland and Germany, with the latter being, at the time, the nearest EU Member State with low alcohol taxes. However, the feasibility of the government’s existing approach to alcohol policy was undermined when the EU (*environmental influences – supranational political organisation*) approved the membership of Estonia, whose alcohol taxes were as low as those in Germany.

On the one hand, economic assessments by the Ministry of Finance predicted that, due to the free movement of goods across EU countries, a considerable increase in Finnish travellers’ import of alcohol from Estonia would take place. They projected decreasing profits for Finnish breweries and other businesses that were dependent on alcohol production and sales (relevant to the *sectoral objectives* of the Ministry of Finance). In an attempt to avoid this scenario, the Ministry of Finance proposed a reduction of alcohol sales taxes by one-third [43]. However, a Health Impact Assessment conducted by the Ministry of Social Affairs and Health on the proposal projected that lower costs for alcohol would lead to increased consumption and related negative health impacts (relevant to the *sectoral objectives* of the Ministry of Social Affairs and Health). The Health Impact Assessment suggested that a tax reduction of 33% would increase the number of heavy drinkers by 200,000 (4% of the total population) and increase alcohol-related deaths by 600 a year [43]. Finally, *political elites* in the government decided to implement the tax reduction.

### Generative mechanisms in Finland: a systems view

Why did economic arguments carry more weight than public health and equity considerations in this case, in spite of *prior ISA experience*? A number of mechanisms involving multiple subsystems and components can help to explain this outcome. Below, we argue that competing *sectoral objectives* between the Ministry of Finance and the Ministry of Social Affairs and Health, i.e. economic and health objectives reflecting distinct interests and ideas, affected the government’s decision-making about alcohol policy in anticipation of Estonia joining the EU. In particular, critical factors included the influence of a strong alcohol lobby (*environmental influences – private sector lobby organisation*), an ideological commitment to neoliberalism since the early 1990s (political ideology) [44] and a concomitant focus on promoting economic growth at the expense of other aims, including social equity (policy agenda) and a non-directive implementation style for HiAP (*HiAP management*).

Joining the EU in 1995 led to liberalisation of the Finnish economy and increased trade, which augmented the role of lobbyists in the political process. By the time Estonia’s membership was approved, a strong lobby advocating for the lowering of alcohol taxes had established itself, even amongst the country’s political executive (*political agenda*) [45], in part through election campaign contributions paid for by the alcohol and retail industries (*environmental influences – private sector lobby organisation*). Mass media campaigns also shaped the political agenda by arousing popular concern over the economic consequences of maintaining high alcohol taxes (*environmental influences – media*). On the other hand, there was comparatively minimal lobbying against the proposal to lower them (*environmental influences – lobby organisation*) [45].

*Political elites* within the government executive were strongly focused on the *sectoral objectives* of the Ministry of Finance, such as securing economic growth and restraining inflation [46]. Our key informants described how economic sectors (such as those concerned with finance, agriculture and industry) were generally less responsive to the results of the Health Impact Assessment, particularly because the *political ideology* of the ruling party favoured economic growth over social objectives such as health equity. The Ministry of Finance, in particular, exhibited resistance to efforts to take into consideration the potential health consequences of their tax reform proposal [46, 47].

Our key informant interviews also suggest that *political elites* in central government did not provide strong leadership in the political process (i.e. arguably a lack of *HiAP management*) to achieve the *sectoral objectives* of the Ministry, which hindered buy-in from sectors for Health 2015. As Vilen [48] notes, widespread popular support for strong tax policies in the 1970s, 1980s, and still in the 1990s had grown weaker by the time Health 2015 was being implemented, so it may have been politically unpopular for *political elites* to advocate for more upstream interventions (concern for *sectoral power*). Vilen [48] also describes how the Minister of Social Affairs and Health and his cabinet were occupied with the ongoing healthcare reform, leaving less time for promoting other issues (competing *sectoral objectives*).

Finally, by using our systems framework to help organise and interpret our data, we might argue that the institutional awareness in the Finnish government of the need for ISA to promote population health and equity coupled with the Health 2015 Strategy were less persuasive on political elites in the Ministry of Finance and elsewhere in the executive than the need to pursue the economic interests of the alcohol lobby in Finland. This assertion can serve as a type of what Merton referred to as “*middle range theory*” ([49], p. 39), which can be explored and refined via a number of critical questions about how and why certain subsystems (and components) enabled economic growth objectives to be prioritised over population health and health equity objectives [50]. For example, what role did the sectoral power of the Ministry of Finance and the Ministry of Social Affairs and Health play in this outcome, particularly in the interaction with the executive subsystem (i.e. a centre-left wing coalition ruling in the era of neoliberalism with strong industry lobby)? Would we expect a different outcome with different political elites in power? Given that the executive subsystem (via the actions of political elites) was more powerful than the intersectoral subsystem (via the HiAP mandate of Health 2015), would a stronger mandate have led to a different outcome (e.g. a law stating that a chief medical officer of health must review Health Impact Assessments and provide oversight of related policy recommendations)?

## Discussion

Our use of systems theory yields several benefits for the implementation of HiAP approaches, including (1) an explicit acknowledgement of the interrelationships between government subsystems and their components involved in HiAP implementation, (2) a framework to contextualise the complex and emergent processes of implementation to help explain how and why system components work together – and under what circumstances – to enable HiAP to succeed or fail, and (3) consideration of how the systems of external influences impact government systems in HiAP implementation. We view this framework as a tool for policy-makers charged with managing implementation processes, and for researchers interested in theorising about HiAP implementation and explaining observations. It will also help those interested in other parts of the HiAP policy process (including design, adoption and evaluation) to understand the complexities of implementation.

Over time, if observations within and across case settings reveal patterns in how system components interact and impact implementation outcomes, general principles of this system may become apparent. For example, we noted that the arguments of the private sector lobby organisations in Finland (to reduce alcohol taxes) ultimately had a negative impact on the implementation of Health 2015 because the ruling party had a political ideology that favoured economic growth over health equity and, thus, opted to not heed the findings of the Health Impact Assessment that was conducted. If this set of relations produces a similar outcome in Finland over time, this may constitute a rationale for introducing governance structures and strategies for managing relationships with private sector stakeholders in the implementation of HiAP. If such outcomes emerge across a variety of settings where HiAP is being implemented, then supranational health organisations, such as WHO, may take the lead in recommending such actions globally.

As local systems are better understood over time, policy-makers can draw on their understanding of inter-relationships amongst components to facilitate what Norman refers to as systems-based “*change strategies*” ([18], p. 870) for health promotion. For example, prior to adopting Health 2015 in Finland, a group of WHO experts identified the reliance on already overstretched civil servants to monitor and evaluate the progress of implementation, in addition to their normal duties, as a weakness of implementing the Finnish Health For All strategy. The idea arose of an Advisory Board for Public Health to function as a collaborative body for intersectoral cooperation [43], and this Board continued to serve as a coordinating mechanism through the implementation of Health 2015. Using the systems framework, the initial challenge can be understood as a problem of workforce capacity for ISA in the intrasectoral subsystem, and the change strategy as responding to that need by introducing a new body for HiAP management in the intersectoral subsystem.

As one example of how researchers can utilise this framework, in HARMONICS, we have drawn on it using a realist science approach to study the social mechanisms of HiAP implementation. Realist science draws on critical realism, which is an ontology that posits the existence of a world outside the observer that remains largely hidden from the observer, like the gears of a clock. Roy Bashkar (a key proponent of critical realism) incorporated systems thinking in his later work [51], and Mingers recently discussed several concepts that belong to systems theory [27], which we have actualised in HARMONICS. For example, the ‘*structure’* of a system can be thought of as the sum of relations within a system, which we have represented as a series of components within subsystems in Fig. 1. ‘*Emergence*’ denotes properties of a system that are not held by any component of the system. This includes the outcomes of HiAP implementation that we focus on in HARMONICS such as sustainability. Finally, realist science generally aims to explain phenomena in ways that allow for complex generative mechanisms. For example, the explanatory case study methodology developed for HARMONICS uses “*context-mechanism-outcomes pattern configurations*” ([5], p. 464) to focus our work on uncovering these generative mechanisms in the course of analysis.

Other realist science methodologies have also been useful for enacting a systems theory approach in HARMONICS. We have drawn on recent work to apply realist science to health system transformation (e.g. [52, 53]), which recommends starting with some “*initial programme theory*” ([53], p. 7) about how an intervention works (including conceptual frameworks and hypotheses), and then using this to guide the articulation of generative mechanisms and to eventually produce a refined theory. Rather than working toward a grand theory that explains all causal instances, the end product here is what sociologists describe as middle range theory, which reflects the causal mechanisms in specific circumstances (i.e. as reflected in a particular database) [49, 50]. Because information about discrete mechanisms can be hard to summarise in a simple manner – and causal attribution difficult to pinpoint – when studying the implementation of HiAP, this systems framework has served as a useful heuristic for developing and testing hypotheses across cases in HARMONICS by revealing components and guiding theory about inter-relationships.

This systems framework is not a panacea for understanding the policy-making process. In particular, we focus mainly on the policy-making process within government at the macro/structural level, with less attention to processes at the micro level, although these are also occurring within the system. While policy-makers can endeavour to plan certain aspects of HiAP implementation, some outcomes will materialise outside the control of individual policy-makers and other social technologists [54]; this is a key quality of complex systems.

We have developed this framework through our experience analysing a series of explanatory case studies of HiAP implementation (e.g. [24]), and theorizing on the topic (e.g. [55, 56]). This framework will be further described and strengthened as we and others continue testing its explanatory power in empirical studies of this topic. Although Table 1 describes some of the influences of extra-governmental systems, it is worth noting that these are likely to be numerous and varied across settings, and this aspect clearly can be further elaborated on. For example, while there are health research systems that create, translate and disseminate knowledge that informs implementation [57, 58], the impact of this knowledge may be of limited utility to policy-makers engaged in HiAP implementation since scientists often focus on one aspect of multi-faceted political problems [59, 60]. Other public, private and ‘third sector’ systems (existing “*between the market and the state*” [61], p. 5) may indirectly influence implementation because their mandates and interests include public health, health equity or social welfare. They may also pursue other objectives that conflict with these goals, like fiscal conservatism or profit.

## Conclusions

Herein, we described a systems framework for the implementation of HiAP that articulates a series of relevant subsystems and components, and then demonstrated the explanatory value of this framework in a case study of the Health 2015 Strategy in Finland. Although policy implementation has been described as a complex process, this systems theory offers an organised view of this process by focusing on certain characteristics of a government and its external environment while uncovering underlying relationships [62]. This contribution is timely as there has been very little application of Systems Theory to health equity interventions, including forms of intersectoral action such as HiAP.

## Abbreviations

HARMONICS: HiAP Analysis using Realist Methods On International Case Studies
HiAP: Health in all Policies
